# The aspect of experience in ultra-triathlon races

**DOI:** 10.1186/s40064-015-1050-3

**Published:** 2015-06-19

**Authors:** Beat Knechtle, Matthias Alexander Zingg, Thomas Rosemann, Christoph Alexander Rüst

**Affiliations:** Facharzt FMH für Allgemeinmedizin, Gesundheitszentrum St. Gallen, Vadianstrasse 26, 9001 St. Gallen, Switzerland; Institute of Primary Care, University of Zurich, Zurich, Switzerland

**Keywords:** Swimming, Cycling, Running, Personal best time

## Abstract

Previous experience seems to be an important predictor for endurance and ultra-endurance performance. The present study investigated whether the number of previously completed races and/or the personal best times in shorter races is more predictive for performance in longer non-stop ultra-triathlons such as a Deca Iron ultra-triathlon. All female and male ultra-triathletes who had finished between 1985 and 2014 at least one Double Iron ultra-triathlon (i.e. 7.6 km swimming, 360 km cycling and 84.4 km running), one Triple Iron ultra-triathlon (i.e. 11.4 km swimming, 540 km cycling and 126.6 km running), one Quintuple Iron ultra-triathlon (i.e. 19 km swimming, 900 km cycling and 221 km running) and one Deca Iron ultra-triathlon (i.e. 38 km swimming, 1,800 km cycling and 422 km running) were identified and their best race times for each distance were recorded. Multiple regression analysis (stepwise, forward selection, p of F for inclusion <0.05, p of F for exclusion >0.1, listwise deletion) was used to determine all variables correlating to overall race time and performance in split disciplines for both Quintuple and Deca Iron ultra-triathlon. The number of finished shorter races (i.e. Double and Triple Iron ultra-triathlon) was not associated with the number of finished longer races (i.e. Quintuple and Deca Iron ultra-triathlon) whereas both split and overall race times correlated to split and overall race times of the longer races with the exception of the swimming split times in Double Iron ultra-triathlon showing no correlation with swimming split times in both Quintuple and Deca Iron ultra-triathlon. In summary, previous experience seemed of importance in performance for longer ultra-triathlon races (i.e. Quintuple and Deca Iron ultra-triathlon) where the personal best times of shorter races (i.e. Double and Triple Iron ultra-triathlon) were important, but not the number of previously finished races. For athletes and coaches, fast race times in shorter ultra-triathlon races (i.e. Double and Triple Iron ultra-triathlon) are more important than a large of number finished races in order to achieve a fast race time in a longer ultra-triathlon (i.e. Quintuple and Deca Iron ultra-triathlon).

## Background

Competing in ultra-endurance races such as ultra-running (Cejka et al. 2014; Hoffman and Wegelin 2009) or ultra-triathlon (Lenherr et al. 2012) is increasing in popularity. An ultra-triathlon is a non-stop long-distance triathlon covering x times the classical Ironman distance triathlon (i.e. 3.8 km swimming, 180 km cycling and 42.195 km running) (Lepers 2008). In the last 30 years between 1985 and 2014, the distances increased from Double Iron ultra-triathlon (i.e. 7.6 km swimming, 360 km cycling and 84.4 km running) to Deca Iron ultra-triathlon (i.e. 38 km swimming, 1,800 km cycling and 422 km running) (Lenherr et al. 2012). However, also race distances of Double Deca Iron ultra-triathlon (i.e. 76 km swimming, 3,600 km cycling and 844 km running) (Knechtle et al. 2014e) and Triple Deca Iron ultra-triathlon (i.e. 114 km swimming, 5,400 km cycling and 1,266 km running) (Knechtle et al. 2014b) have been held.

Previous experience seems to be very important in ultra-endurance performance such as long-distance triathlon (Gilinsky et al. 2014; Gulbin and Gaffney 1999; Herbst et al. 2011; Knechtle et al. 2014a, b). The number (Herbst et al. 2011) and the personal best times achieved in previous races (Gilinsky et al. 2014; Gulbin and Gaffney 1999; Knechtle et al. 2010a, 2011a, b, c, 2012) seemed both important for a fast race time in a longer triathlon race. For example, for triathletes competing in a half-Ironman, the best predictors of race performance were age, previous best half-Ironman time, goal time, and importance of reaching this goal (Gilinsky et al. 2014). Are the previous personal best times and/or the numbers of completed races accurate predictors of ultra-triathlon performance?

Especially for longer triathlon distances (e.g. Ironman triathlon and longer), the personal best time of a shorter race was predictive for the race time in a longer race. For Ironman triathletes, the personal best time in an Olympic distance triathlon was an important predictor variable (Gulbin and Gaffney 1999; Knechtle et al. 2010b; Rüst et al. 2011, 2012a). For Triple Iron ultra-triathletes (i.e. 11.4 km swimming, 540 km cycling, and 126.6 km running), the personal best time in an Ironman triathlon was positively and highly significantly related to overall race time (Knechtle et al. 2011d).

Recent case and field studies investigating athletes competing in ultra-triathlons of 30 times the Ironman distance and longer assumed that previous experience seemed the most important predictor for a successful outcome (Knechtle et al. 2014a, b). An athlete completed 33 Ironman triathlons during 33 days in a self-paced race with minor daily variations over time (i.e. even pacing) in both split and overall race times (Knechtle et al. 2014a). This performance was most probably due to the high experience of the athlete with many completed previous triathlons from Olympic distance triathlon to Deca Iron ultra-triathlon (Knechtle et al. 2014a). Similarly, athletes who completed for the first time in history a race covering 30 Ironman triathlons in 30 days showed an even pacing with no changes in split, transition and overall race times (Knechtle et al. 2014b). The evaluation of previous experience in the successful finishers showed that these athletes had finished prior to the investigated event very long ultra-triathlons up to the Double Deca Iron ultra-triathlon and had relatively fast personal best times in both Double and Triple Iron ultra-triathlon (Knechtle et al. 2014b).

For ultra-triathlons, athletes who had competed in Double and Triple Iron ultra-triathlon showed a significant correlation between swimming, cycling and overall race time with the corresponding times in a Deca Iron ultra-triathlon (Lepers et al. 2011). Running performance in Double and Triple Iron ultra-triathlon was, however, not related to performance in Deca Iron ultra-triathlon (Lepers et al. 2011). The association between personal best times in shorter ultra-triathlon distances with the longest ultra-triathlon distance is known (Herbst et al. 2011; Lepers et al. 2011). However, also the number of previously finished races seems to be of importance in ultra-endurance athletes such as ultra-marathoners (Hoffman and Parise 2015; Knechtle et al. 2014c). For female and male runners competing in 161-km ultramarathons in North America from 1974 through 2010 with at least three finishes, the number of finished races was inversely associated with overall race time (Hoffman and Parise 2015). Also in ultra-marathoners competing in time-limited ultra-marathons held from 6 h to 10 days during from 1975 to 2013, athletes improved race performance with increasing number of successful finishes (Knechtle et al. 2014c).

For ultra-triathlons, one study showed that both the number of and the performance in shorter races are important for the performance in a longer race such as a Deca Iron ultra-triathlon. The number of finished races and the personal best time in a Triple Iron ultra-triathlon was related to overall race time in a Deca Iron ultra-triathlon (Herbst et al. 2011). However, an analysis whether both the number of finished races and/or the previous best times are related to performance in longer ultra-triathlon races is missing. We therefore tested the hypothesis that both the number of finished races and the personal best time in a shorter race would be important for the performance in a longer race by investigating potential associations between the numbers and the split and overall race times of shorter (i.e. Double, Triple and Quintuple Iron ultra-triathlon) with the corresponding number and times of longer races (i.e. Deca Iron ultra-triathlon).

## Methods

### Ethics

All procedures used in the study were approved by the Institutional Review Board of Kanton St. Gallen, Switzerland with a waiver of the requirement for informed consent of the participants given the fact that the study involved the analysis of publicly available data.

### Data collection

All race results for ultra-triathlons held between 1985 and 2014 were collected by one of the authors. Data were obtained in electronic form from the official race websites or in printed form for earlier editions by mail from the race directors. All female and male athletes who had ever finished at least one Double, one Triple, one Quintuple and one Deca Iron ultra-triathlon were identified and their best split and overall race times for each distance were recorded.

### Statistical analysis

The relationship between swimming, cycling, running and overall race times of the four ultra-distances was analyzed using linear regression analysis. Multiple regression analysis (stepwise, forward selection, p of F for inclusion <0.05, p of F for exclusion >0.1, listwise deletion) was used to determine all variables (i.e. split and overall race times) correlating to split and overall race times for both Quintuple and Deca Iron ultra-triathlon. The investigated variables were experience and performance in finished ultra-triathlons, where experience was defined as the number of previously finished Double Iron ultra-triathlons and Triple Iron ultra-triathlons for the performance in Quintuple Iron ultra-triathlon and the number of previously finished Double, Triple, and Quintuple Iron ultra-triathlons for the performance in Deca Iron ultra-triathlon. Regarding the performance for each split discipline and for overall race time, the previous best performance in Double and Triple Iron ultra-triathlon was considered for the analysis of the best Quintuple Iron ultra-triathlon performance and the best performances in previously finished Double, Triple, and Quintuple Iron ultra-triathlons was considered for the analysis of best Deca Iron ultra-triathlon performance. The absence of autocorrelation was tested using a Durbin–Watson test. Heteroscedasticity was tested using a diagram showing the standardized values for y on the x-axis and the standardized residuals on the y-axis. Multicollinearity was tested by calculating tolerance value as well as variance inflation factor (VIF). Statistical analyses were performed using IBM SPSS Statistics (Version 22, IBM SPSS, Chicago, IL, USA) and GraphPad Prism (Version 6.01, GraphPad Software, La Jolla, CA, USA). Significance was accepted at p < 0.05 (two-sided for *t* tests). Data in the text and figures are given as mean ± standard deviation (SD).

## Results

### The number of finished races

Between 1985 and 2014, a total of 1,971, 1,075, 54 and 131 finishes were recorded for Double, Triple, Quintuple and Deca Iron ultra-triathlon, respectively. A total of 26 athletes had finished a Quintuple Iron ultra-triathlon and at least one Double and one Triple Iron ultra-triathlon. The mean numbers of successful finishes were 7.2 ± 10.5 Double and 6.1 ± 7.1 Triple Iron ultra-triathlons. Among those athletes who finished more than one Triple and one Double Iron ultra-triathlon, the number of finished Double (*r* = −0.14, p = 0.24) and Triple (*r* = −0.21, p = 0.15) Iron ultra-triathlons showed no relationship to the number of finished Quintuple Iron ultra-triathlons. Considering the Deca Iron ultra-triathlon distance, a total of 15 athletes had also finished at least one Double, one Triple and one Quintuple Iron ultra-triathlon. The mean numbers of successful finishes were 9.5 ± 12.3 Double, 7.1 ± 7.3 Triple and 1.3 ± 0.5 Quintuple Iron ultra-triathlons. The number of finished Double (*r* = −0.10, p = 0.35), Triple (*r* = −0.26, p = 0.17) and Quintuple (*r* = 0.03, p = 0.45) Iron ultra-triathlons was not correlated to the number of finished Deca Iron ultra-triathlons.

### Performance in previous finished races

**Figure 1:**
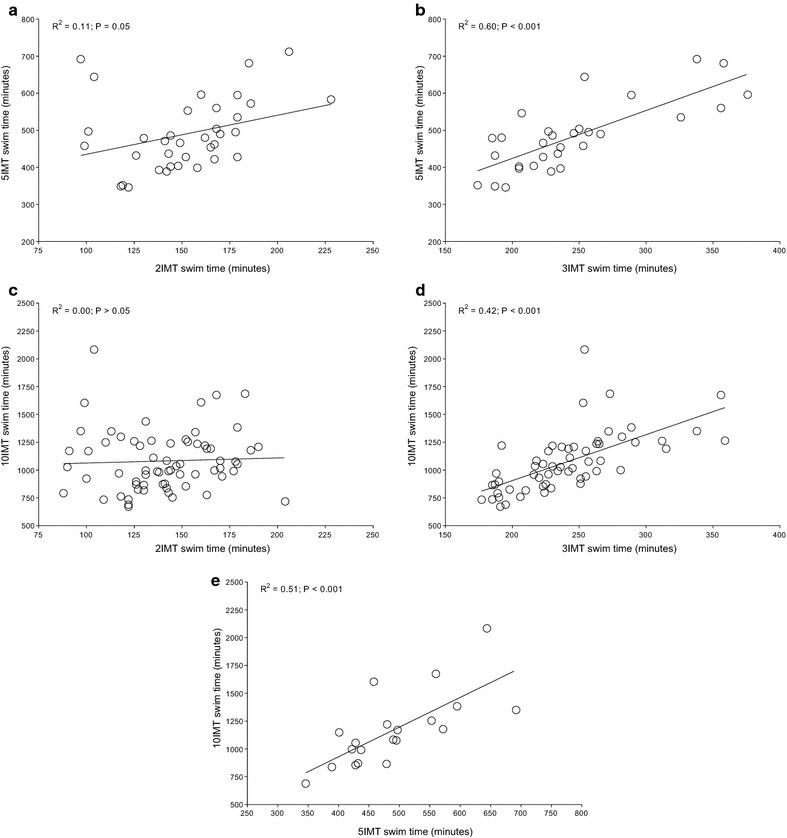
The correlation in swimming split times between Double and Quintuple (**a**), Triple and Quintuple (**b**), Double and Deca (**c**), Triple and Deca (**d**) and Quintuple and Deca Iron ultra-triathlon (**e**).

**Figure 2:**
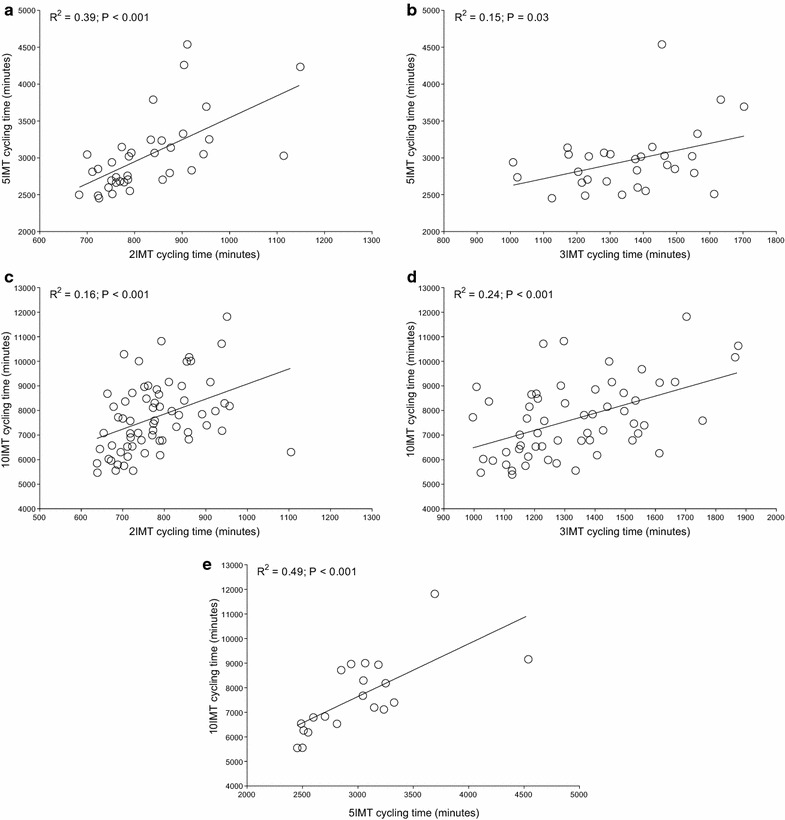
The correlation in cycling split times between Double and Quintuple (**a**), Triple and Quintuple (**b**), Double and Deca (**c**), Triple and Deca (**d**) and Quintuple and Deca Iron ultra-triathlon (**e**).

**Figure 3:**
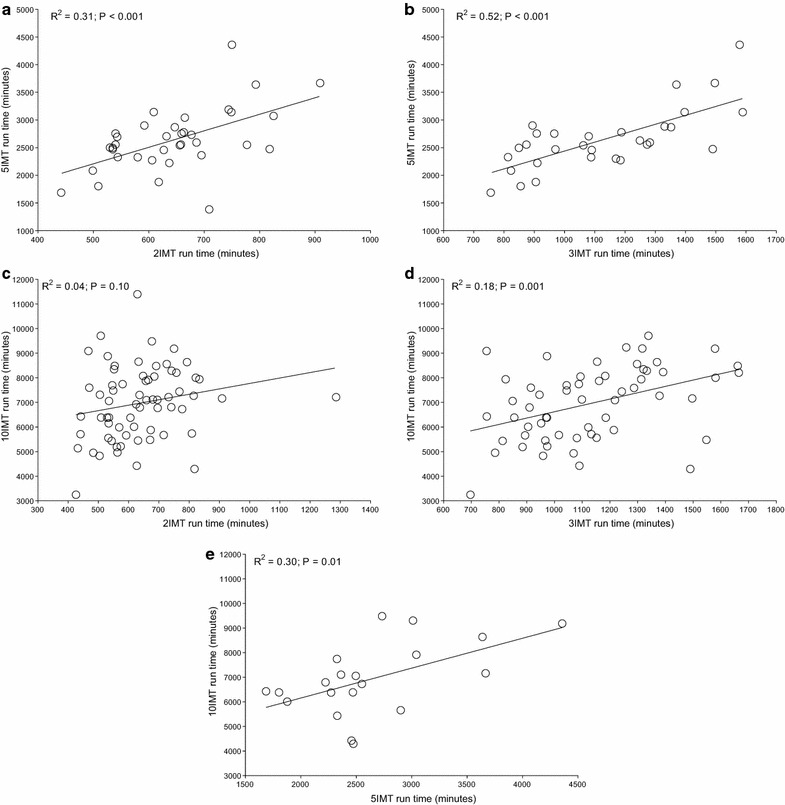
The correlation in running split times between Double and Quintuple (**a**), Triple and Quintuple (**b**), Double and Deca (**c**), Triple and Deca (**d**) and Quintuple and Deca Iron ultra-triathlon (**e**).

**Figure 4:**
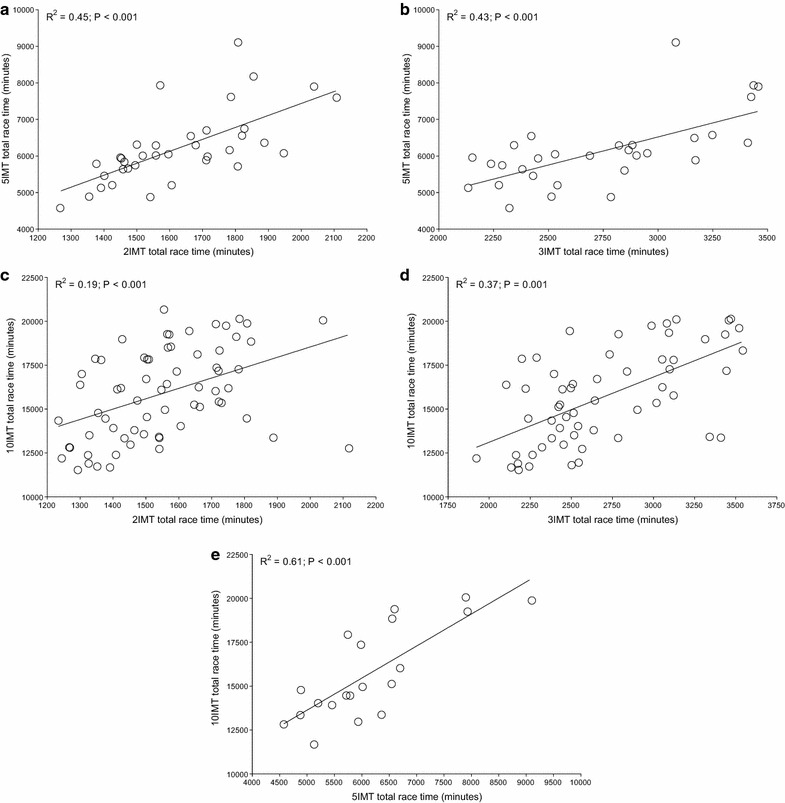
The correlation in overall race times between Double and Quintuple (**a**), Triple and Quintuple (**b**), Double and Deca (**c**), Triple and Deca (**d**) and Quintuple and Deca Iron ultra-triathlon (**e**).

For both Quintuple and Deca Iron ultra-triathlon, all split times correlated to overall race times (Table 1). In detail, swimming split times in Triple Iron ultra-triathlon correlated to split times in both Quintuple and Deca Iron ultra-triathlon and swimming split times in Quintuple Iron correlated to swimming split times in Deca Iron ultra-triathlon (Figure 1). Swimming split times in Double Iron ultra-triathlon, however, showed no correlation with swimming split times in both Quintuple and Deca Iron ultra-triathlon. For cycling (Figure 2) and running (Figure 3), split times in the shorter distances correlated to split times in the longer distances. Also, overall race times in the shorter distances were associated with overall race times in the longer distances (Figure 4).

**Table 1:** Results of the multiple regression analysis for Quintuple and Deca Iron ultra-triathlons

	ß	t	*r* ^*2*^	p	F	Durbin–Watson	T	VIF
Quintuple Iron								
Swimming	0.804	6.631	0.632	<0.001	43.969	2.537	1.000	1.000
Cycling	0.501	2.837	0.220	0.0090	8.0510	1.476	1.000	1.000
Running	0.729	5.216	0.512	<0.0001	27.209	1.946	1.000	1.000
Overall race time	0.666	4.370	0.420	<0.0001	19.13	2.116	1.000	1.000
Deca Iron								
Swimming	0.736	3.925	0.507	0.0020	15.403	1.869	1.000	1.000
Cycling	0.718	3.716	0.478	0.0030	13.805	2.731	1.000	1.000
Running	0.588	2.624	0.296	0.0210	6.8850	1.320	1.000	1.000
Overall race time	0.833	5.427	0.670	<0.0001	29.456	1.827	1.000	1.000

*ß* regression coefficient, *F* F-value, *t* Student’s t distribution, *T* tolerance, *VIF* variance inflation factor.

Overall race time in Deca Iron ultra-triathlon might be partially predicted by the following equations: (1) Deca Iron ultra-triathlon race time (min) (*r*^2^ = 0.10) = 17,191 + 2.799 × Double Iron ultra-triathlon race time (min) − 1.821 × Triple Iron ultra-triathlon race time (min) − 0.03039 × Quintuple Iron ultra-triathlon race time (min), (2) Deca Iron ultra-triathlon race time (min) (*r*^2^ = 0.02) = 12,104 + 1.433 × Double Iron ultra-triathlon race time (min) + 0.466 × Triple Iron ultra-triathlon race time (min), (3) Deca Iron ultra-triathlon race time (min) (*r*^2^ = 0.02) = 13,552 + 1.389 × Double Iron ultra-triathlon race time (min), (4) Deca Iron ultra-triathlon race time (min) (*r*^2^ = 0.00) = 15,015 + 0.3965 × Triple Iron ultra-triathlon race time (min), (5) Deca Iron ultra-triathlon race time (min) (*r*^2^ = 0.00) = 16,678 + 0.001833 × Quintuple Iron ultra-triathlon race time (min). For Quintuple Iron ultra-triathlon, race time might be partially predicted by the equations: (1) Quintuple Iron ultra-triathlon race time (min) (*r*^2^ = 0.04) = 8,892 − 0.2986 × Double Iron ultra-triathlon race time (min) − 0.6211 × Triple Iron ultra-triathlon race time (min), (2) Quintuple Iron ultra-triathlon race time (min) (*r*^2^ = 0.01) = 7,318 − 0.4593 × Double Iron ultra-triathlon race time (min) or (3) Quintuple Iron race time (min) (*r*^2^ = 0.01) = 8.480 − 0.6677 × Triple Iron ultra-triathlon race time (min).

## Discussion

The present study investigated a potential relationship between the number of previously finished ultra-triathlons and the personal best times with the overall race times in even longer race distances such as a Quintuple and a Deca Iron ultra-triathlon. The most important findings were, first, the number of finished shorter races was not associated with the number of finished longer races and, second, split and overall race times correlated to split and overall race times of longer races with the exception of the swimming split times in Double Iron ultra-triathlon, which showed no correlation with swimming split times in both Quintuple and Deca Iron ultra-triathlon.

### No association between the number of completed races and performance

A first important finding was that the number of previously completed races showed no association with the achieved best times in the longer non-stop race distances (i.e. Quintuple and Deca Iron ultra-triathlon). In a study investigating the performance of Deca Iron ultra-triathletes competing in a race consisting of ten times an Ironman triathlon within 10 days, both the number of finished Triple Iron triathlons and the personal best time in a Triple Iron triathlon were related to race time (Herbst et al. 2011). However, the number of previously finished races seems not predictive in these athletes as it also been reported for other endurance disciplines. For example, in male 24-h ultra-marathoners, the number of previously finished 24-h races was not related to race performance (Knechtle et al. 2011a). Similarly, the number of finished marathons and 100-km ultra-marathons before a 24-h run was not associated with the achieved distance (Knechtle et al. 2009, 2011a). Also for women, the number of finished half-marathons was not related to half-marathon race time (Knechtle et al. 2011f). In ultra-cyclists competing in the ‘Swiss Cycling Marathon’ (Knechtle et al. 2011g) and in the ‘Swiss Bike Master’ (Knechtle et al. 2011h), the number of previously finished races was not related to race time. Also when long-distance triathlons were investigated, both the number of completed Ironman and Triple Iron ultra-triathlons were not related to race time in a Triple Iron ultra-triathlon (Knechtle et al. 2011d). With the present findings we must assume that a high number of previously finishes is not a guarantee for a faster finish in an ultra-distance race in contrast to the achieved personal best times in previous races.

### Split and overall race times correlate to performance in longer race distances

A second important finding was that split and overall race times of the shorter distances correlated to split and overall race times in the longer race distances. However, swimming split times in Double Iron ultra-triathlon showed no correlation with swimming split times in Quintuple and Deca Iron ultra-triathlon. These findings are different to the findings of Lepers et al. (2011) where split times in swimming and cycling in Double and Triple Iron ultra-triathlon correlated to split times in Deca Iron ultra-triathlon, but not running split times. Although Lepers et al. (2011) also identified all triathletes who finished at least one ultra-triathlon in each of three distances (i.e. Double Iron, Triple Iron, and Deca Iron ultra-triathlon) the disparate findings might be explained that we also included the Quintuple Iron. Furthermore, Lepers et al. (2011) considered only a 5-year period compared to our 30-year period from 1985 to 2014.

The present findings that split times in shorter races in both running and cycling, but not in swimming, correlated to split times in longer races seems reasonable. Studies investigating the importance of split times for overall race times in Triple Iron ultra-triathlon showed that cycling and running split times, but not swimming split times, correlated to overall race time (Knechtle et al. 2007a; Knechtle and Kohler 2009). Also for shorter triathlon distances as for example the ‘Muncie Endurathon’ (i.e. 1.2 mile swim, 56 mile cycle, 13.1 mile run) time spent in running and cycling during the race was significantly related to overall race time whereas swimming split time was not related to overall race time (Dengel et al. 1989).

We performed different multiple regression analyses to find the best equation to predict Quintuple and Deca Iron ultra-triathlon races times based upon previous split and race times. The best equations were for Quintuple Iron ultra-triathlon (min) (*r*^2^ = 0.04) = 8,892 − 0.2986 × Double Iron ultra-triathlon race time − 0.6211 × Triple Iron ultra-triathlon race time and for Deca Iron ultra-triathlon (min) (*r*^2^ = 0.10) = 17,191 + 2.799 × Double Iron ultra-triathlon race time (min) − 1.821 × Triple Iron ultra-triathlon race time (min) − 0.03039 × Quintuple Iron ultra-triathlon race time (min). Although the *r*^2^-values were very low, equations with less race times (e.g. only one shorter race distance) showed even lower *r*^2^-values. Obviously, athletes who had completed more races over shorter distances (i.e. a Double and a Triple Iron ultra-triathlon instead of only one distance) than the intended longer distance seemed better prepared for the longer distance. Lepers et al. (2011) presented the equation for Deca Iron ultra-triathlon race time (min) (*r*^2^ = 0.36) = 236 + 3.57 × Triple Iron ultra-triathlon race time (min) with a considerably higher *r*^2^-value. The disparate findings might be explained by the different size of the samples. However, in both instances, the *r*^2^-values were very low and other predictor variables such as anthropometric (Knechtle et al. 2008) or training (Knechtle et al. 2011e) characteristics might be of higher importance to predict ultra-triathlon race time.

Most probably, factors such as motivation (De Ioannon et al. 2015), support crew (Black et al. 2012) and sleep deprivation (Lahart et al. 2013) are higher in a longer ultra-triathlon (i.e. Quintuple and Deca Iron ultra-triathlon) compared to a shorter ultra-triathlon (i.e. Double and Triple Iron ultra-triathlon).

This study is limited due to the fact that aspects of age (Knechtle et al. 2014d), training (Knechtle et al. 2010c), anthropometry (Knechtle et al. 2007b), nutrition (Jeukendrup 2011; Rüst et al. 2012b), motivation (De Ioannon et al. 2015), support crew (Black et al. 2012), pacing (Herbst et al. 2011; Wu et al. 2015), environmental and geographic conditions (Wegelin and Hoffman 2011), and nationality (Rüst et al. 2014) were not considered. These variables might have had an influence on the race times of these athletes.

### Practical applications

For triathletes intending to compete in a very long ultra-triathlon (i.e. Quintuple and Deca Iron ultra-triathlon), fast race times in shorter race distances (i.e. Double and Triple Iron ultra-triathlon) seemed important for a fast race time. However, there seems no need for a high number of race finishes prior to the intended race. Since marathon and Olympic distance triathlon are predictive for Ironman triathlon, we may assume that also a fast Ironman race time might be predictive for a fast ultra-triathlon race time. Future studies might include the personal best time in an Ironman triathlon to predict ultra-triathlon performance.

## Conclusion

To summarize, in ultra-triathletes competing in races from Double Iron to Deca Iron ultra-triathlon, the previous experience in shorter races is of importance for a fast race time in longer ultra-triathlon races (i.e. Quintuple and Deca Iron ultra-triathlon). The personal best times of shorter races (i.e. Double and Triple Iron ultra-triathlon) seemed important, but not the number of previously finished races. For athletes and coaches, fast race times in shorter ultra-triathlon races (i.e. Double and Triple Iron ultra-triathlon) are more important than a large number of finished races in order to achieve a fast race time in a longer ultra-triathlon (i.e. Quintuple and Deca Iron ultra-triathlon).

## References

[CR1] Black KE, Skidmore PM, Brown RC (2012). Energy intakes of ultraendurance cyclists during competition, an observational study. Int J Sport Nutr Exerc Metab.

[CR2] Cejka N, Rüst CA, Lepers R, Onywera V, Rosemann T, Knechtle B (2014). Participation and performance trends in 100-km ultra-marathons worldwide. J Sports Sci.

[CR3] De Ioannon G, Cibelli G, Mignardi S, Antonelli A, Capranica L, Piacentini MF (2015). Pacing and mood changes while crossing the Adriatic Sea from Italy to Albania: a case study. Int J Sports Physiol Perform.

[CR4] Dengel DR, Flynn MG, Costill DL, Kirwan JP (1989). Determinants of success during triathlon competition. Res Q Exerc Sport.

[CR5] Gilinsky N, Hawkins KR, Tokar TN, Cooper JA (2014). Predictive variables for half-Ironman triathlon performance. J Sci Med Sport.

[CR6] Gulbin JP, Gaffney PT (1999). Ultraendurance triathlon participation: typical race preparation of lower level triathletes. J Sports Med Phys Fit.

[CR7] Herbst L, Knechtle B, Lopez CL, Andonie JL, Fraire OS, Kohler G (2011). Pacing strategy and change in body composition during a Deca Iron Triathlon. Chin J Physiol.

[CR8] Hoffman MD, Parise CA (2015). Longitudinal assessment of the effect of age and experience on performance in 161-km ultramarathons. Int J Sports Physiol Perform.

[CR9] Hoffman MD, Wegelin JA (2009). The Western States 100-Mile Endurance Run: participation and performance trends. Med Sci Sports Exerc.

[CR10] Jeukendrup AE (2011). Nutrition for endurance sports: marathon, triathlon, and road cycling. J Sports Sci.

[CR11] Knechtle B, Kohler G (2009). Running performance, not anthropometric factors, is associated with race success in a Triple Iron Triathlon. Br J Sports Med.

[CR12] Knechtle B, Duff B, Amtmann G, Kohler G (2007). Cycling and running performance, not anthropometric factors, are associated with race performance in a Triple Iron Triathlon. Res Sports Med.

[CR13] Knechtle B, Knechtle P, Andonie JL, Kohler G (2007). Influence of anthropometry on race performance in extreme endurance triathletes: world challenge Deca Iron Triathlon 2006. Br J Sports Med.

[CR14] Knechtle B, Schwanke M, Knechtle P, Kohler G (2008). Decrease in body fat during an ultra-endurance triathlon is associated with race intensity. Br J Sports Med.

[CR15] Knechtle B, Wirth A, Knechtle P, Zimmermann K, Kohler G (2009). Personal best marathon performance is associated with performance in a 24-h run and not anthropometry or training volume. Br J Sports Med.

[CR16] Knechtle B, Wirth A, Knechtle P, Rosemann T (2010). Training volume and personal best time in marathon, not anthropometric parameters, are associated with performance in male 100-km ultrarunners. J Strength Cond Res.

[CR17] Knechtle B, Wirth A, Baumann B, Knechtle P, Rosemann T (2010). Personal best time, percent body fat, and training are differently associated with race time for male and female ironman triathletes. Res Q Exerc Sport.

[CR18] Knechtle B, Wirth A, Rosemann T (2010). Predictors of race time in male Ironman triathletes: physical characteristics, training, or prerace experience?. Percept Mot Skills.

[CR19] Knechtle B, Knechtle P, Rosemann T, Lepers R (2011). Personal best marathon time and longest training run, not anthropometry, predict performance in recreational 24-hour ultrarunners. J Strength Cond Res.

[CR20] Knechtle B, Knechtle P, Rosemann T, Senn O (2011). Personal best time and training volume, not anthropometry, is related to race performance in the ‘Swiss Bike Masters’ mountain bike ultramarathon. J Strength Cond Res.

[CR21] Knechtle B, Knechtle P, Rosemann T, Senn O (2011). What is associated with race performance in male 100-km ultra-marathoners––anthropometry, training or marathon best time?. J Sports Sci.

[CR22] Knechtle B, Knechtle P, Rosemann T, Senn O (2011). Personal best time, not anthropometry or training volume, is associated with total race time in a triple iron triathlon. J Strength Cond Res.

[CR23] Knechtle B, Knechtle P, Rüst CA, Rosemann T (2011). A comparison of anthropometric and training characteristics of Ironman triathletes and Triple Iron ultra-triathletes. J Sports Sci.

[CR24] Knechtle B, Knechtle P, Barandun U, Rosemann T (2011). Anthropometric and training variables related to half-marathon running performance in recreational female runners. Phys Sportsmed.

[CR25] Knechtle B, Knechtle P, Rüst CA, Rosemann T, Lepers R (2011). Finishers and nonfinishers in the ‘Swiss Cycling Marathon’ to qualify for the ‘Race Across America’. J Strength Cond Res.

[CR26] Knechtle B, Knechtle P, Rüst CA, Rosemann T, Lepers R (2012). Age, training, and previous experience predict race performance in long-distance inline skaters, not anthropometry. Percept Mot Skills.

[CR27] Knechtle B, Rüst CA, Rosemann T, Martin N (2014). 33 Ironman triathlons in 33 days––a case study. Springerplus.

[CR28] Knechtle B, Rosemann T, Lepers R, Rüst CA (2014). A comparison of performance of Deca Iron and Triple Deca Iron ultra-triathletes. Springerplus.

[CR29] Knechtle B, Valeri F, Zingg MA, Rosemann T, Rüst CA (2014). What is the age for the fastest ultra-marathon performance in time-limited races from 6 h to 10 days?. Age (Dordr).

[CR30] Knechtle R, Rüst CA, Rosemann T, Knechtle B (2014). The best triathletes are older in longer race distances––a comparison between Olympic, Half-Ironman and Ironman distance triathlon. Springerplus.

[CR31] Knechtle B, Zingg MA, Rosemann T, Rüst CA (2014). Sex difference in top performers from Ironman to double deca iron ultra-triathlon. Open Access J Sports Med.

[CR32] Lahart IM, Lane AM, Hulton A, Williams K, Godfrey R, Pedlar C (2013). Challenges in maintaining emotion regulation in a sleep and energy deprived state induced by the 4800km ultra-endurance bicycle race; The Race Across AMerica (RAAM). J Sports Sci Med.

[CR33] Lenherr R, Knechtle B, Rüst CA, Rosemann T, Lepers R (2012). From Double Iron to Double Deca Iron ultra-triathlon––a retrospective data analysis from 1985 to 2011. Phys Cult Sport Stud Res.

[CR34] Lepers R (2008). Analysis of Hawaii ironman performances in elite triathletes from 1981 to 2007. Med Sci Sports Exerc.

[CR35] Lepers R, Knechtle B, Knechtle P, Rosemann T (2011). Analysis of ultra-triathlon performances. Open Access J Sports Med.

[CR36] Rüst CA, Knechtle B, Knechtle P, Rosemann T, Lepers R (2011). Personal best times in an Olympic distance triathlon and in a marathon predict Ironman race time in recreational male triathletes. Open Access J Sports Med.

[CR37] Rüst CA, Knechtle B, Wirth A, Knechtle P, Ellenrieder B, Rosemann T (2012). Personal best times in an Olympic distance triathlon and a marathon predict an ironman race time for recreational female triathletes. Chin J Physiol.

[CR38] Rüst CA, Knechtle B, Knechtle P, Rosemann T (2012). Higher prevalence of exercise-associated hyponatremia in triple iron ultra-triathletes than reported for ironman triathletes. Chin J Physiol.

[CR39] Rüst CA, Knechtle B, Knechtle P, Lepers R, Rosemann T, Onywera V (2014). European athletes dominate performances in Double Iron ultra-triathlons––a retrospective data analysis from 1985 to 2010. Eur J Sport Sci.

[CR40] Wegelin JA, Hoffman MD (2011). Variables associated with odds of finishing and finish time in a 161-km ultramarathon. Eur J Appl Physiol.

[CR41] Wu SS, Peiffer JJ, Brisswalter J, Nosaka K, Lau WY, Abbiss CR (2015). Pacing strategies during the swim, cycle and run disciplines of sprint, Olympic and half-Ironman triathlons. Eur J Appl Physiol.

